# Use of complement C5-inhibitor eculizumab in patients with infection-associated hemolytic uremic syndrome – a case-series report

**DOI:** 10.1186/s12887-025-05546-3

**Published:** 2025-03-11

**Authors:** Petra Varga, Erika Biró, Andrea Berkes, Erzsébet Lakatos, Edit Szikszay, Zoltán Prohászka, Tamás Szabó

**Affiliations:** 1 Institute of Pediatrics, Faculty of Medicine, University of Debrecen, Nagyerdei Krt 98, 4028 Debrecen, Hungary; 2 Department of Internal Medicine and Hematology, Semmelweis University, Szentkirály Str. 46, 1088 Budapest, Hungary

**Keywords:** Hemolytic uremic syndrome, Infection-associated HUS, STEC-HUS, SP-HUS, Eculizumab, CNS involvement

## Abstract

**Background:**

Hemolytic uremic syndrome (HUS), characterized by the triad of microangiopathic hemolytic anemia, thrombocytopenia and acute kidney injury (AKI), remains a leading cause of pediatric AKI. The complement system has a crucial role in the pathogenesis of atypical hemolytic uremic syndrome (aHUS) and eculizumab (ECZ) was approved as standard of care for its treatment. The two widely characterized forms of infection-associated HUS are Shiga toxin-producing E. coli (STEC)-HUS and Streptococcus pneumoniae-associated (SP)-HUS. Extrarenal manifestations such as central nervous system (CNS) involvement occur approximately in 20% of the cases and are accompanied by higher mortality. Abnormalities of the alternative complement pathway may also contribute to the development of both STEC-HUS and SP-HUS, offering a potential treatment option for complement C5 inhibition. Beyond best supportive care as standard therapeutic approach, ECZ has been successfully used in both STEC-HUS and SP-HUS patients.

We provide further support that early use of ECZ for infection-associated HUS with severe clinical manifestation and abnormal complement-activation profile may be an effective therapeutic approach.

**Case presentation:**

We report on three children (median age: 2 years, range: 2–10 years) diagnosed with infection-associated HUS treated with complement C5-inhibitor ECZ. All three patients were treated with ECZ and had excellent outcome. We retrospectively analyzed the clinical course, laboratory data and outcome of children with infection-associated HUS treated with ECZ.

**Conclusion:**

In accordance with previous observations ECZ is an efficacious therapeutic choice in severe HUS patients with multiorgan involvement. A detailed complement activation profile, especially sC5b-9, is useful to indicate ECZ administration.

## Background

Hemolytic uremic syndrome (HUS) is charaterized by the triad of microangiopathic hemolytic anemia, thrombocytopenia and acute kidney injury (AKI) [1]. Infection is the most common cause of HUS as Shiga-like toxin (verotoxin) producing bacteria, including Shiga toxin-producing enterohaemorrhagic Escherichia coli (STEC), mostly E. coli serotype 0157:H7 or Shigella dysenteriae type 1 are responsible for 90% of all HUS cases [2, 3]. The acute phase of STEC-HUS is severe, with a mortality rate of up to 5% while it may reach up to 20% when presenting with neurological involvement. At least 50% of pediatric HUS patients require dialysis and 30% survive with long-term renal sequelae [3–6]. Streptococcus pneumoniae-associated HUS (SP-HUS) represents approximately 5–15% of all HUS cases, of which long-term kidney outcome seems to be similar to STEC-HUS [7]. Higher mortality rate has been reported in 11–16% of SP-HUS patients, where neuraminidase-induced endothelium damage leads to the activation of the complement system and thrombotic microangiopathy (TMA) [8, 9].

A recent publication reports that a working group of specialists in thrombotic microangiopathies was convened to reclassify TMA on the basis of the main mechanism/etiology of the underlying disease. Thus, infection-associated TMA is listed as a separate entity, with STEC, pneumococcus, viral infection and sepsis as its background. This approach was believed to best support potential treatment methods and utilization of more tailored therapies [10].

Current management of both STEC-HUS and SP-HUS is based on supportive care, including fluid resuscitation, fluid- and electrolyte balance control, blood pressure control, continuous kidney replacement therapy (CKRT) and hematological support. While there is no validated specific therapy, the role of complement dysregulation has been established in either STEC-HUS or other infection-related cases of HUS, suggesting that eculizumab (ECZ) (trade name Soliris; Alexion Pharmaceuticals) may be a useful therapy [4, 8]. ECZ, a humanized monoclonal C5 antibody inhibits terminal complement complex formation. ECZ has been approved both by the European Medicines Agency (EMA) and by the US Food and Drug Administration (FDA) in September 2011 for the treatment of atypical hemolytic uremic syndrome (aHUS) as standard of care, found to be effective in preventing progression to end-stage renal disease [11]. The role of complement activation (AP) has been established in STEC-HUS, although the exact mechanism is still unclear [12]. Previous case reports and cohort studies as well as a current meta-analysis have demonstrated potential benefits of using ECZ, and described convincing clinical improvement after treatment with ECZ in severe STEC-HUS with progressive neurological involvement [13]. None of these studies was randomized or blinded [12, 14–16]. A recent randomized, controlled study revealed no convincing benefit of ECZ used in the acute phase of all STEC-HUS cases, however significantly better long-term kidney outcome was observed in the treated group [17]. Still, decision about the potential use of ECZ requires a multidisciplinary background and judgement is often made on the basis of clinical parameters of disease progression. Conventional complement serology studies (C3, C4, CH50) may not be informative, as often only marginal changes can be detected and kidney biopsy is not performed when diagnosis is otherwise confirmed. An extended complement activation panel may provide further data about the activation profile and help guide clinical decision [18, 19].

Genetic analysis of the complement regulation cascade is not part of the routine clinical investigation in infection-associated HUS types, even though it may provide additional information about long-term prognosis [19].

Herein, we are presenting a series of three children diagnosed with infection-associated HUS treated with complement C5-inhibitor ECZ.

## Case presentations

We retrospectively reviewed three infection-associated HUS cases treated with complement C5-inhibitor ECZ in our institute (Institute of Pediatrics, University of Debrecen, Hungary). HUS was defined as hemolytic anemia, thrombocytopenia and acute kidney injury (AKI) caused by TMA. AKI was defined by KDIGO criteria [20]. The presence of confirmed infection (stool culture, blood culture and/or polymerase chain reaction (PCR) was necessary for the infection-associated HUS diagnosis. ECZ therapy was indicated in patients with signs of alternative complement pathway (AP) activation.

We documented demographics (gender, age, body weight (BW), height, body mass index (BMI), microbiological data (primary site of infection, results of bacterial cultures, PCR studies), laboratory panel (haemoglobin, platelet, creatinine, urea, LDH, fragmentocytes, haptoglobin, CRP, direct Coombs test) including complement factors and activation (ADAMTS13 metalloproteinase activity, CH50, APH50, C3, C4, CFH, CFI, CFB, C1q, sC5b-9), results of genetic analysis (mutation of CFH, CFI, CD46, C3, CFB, THBD, CFHR5, DGKE) (Department of Internal Medicine and Hematology, Semmelweis University, Budapest, Hungary). As well as major clinical data including duration and modality of kidney replacement therapy, radiological investigation and administration of ECZ and follow-up parameters (3–6 months) included estimated GFR (eGFR) using the Schwartz formula, proteinuria defined by protein-creatinine ratio from spot urine, presence of hematuria and arterial hypertension (defined as blood pressure values above the 95th percentile in at least three individual measurements [21].

### Patient 1

**Fig. 1:**
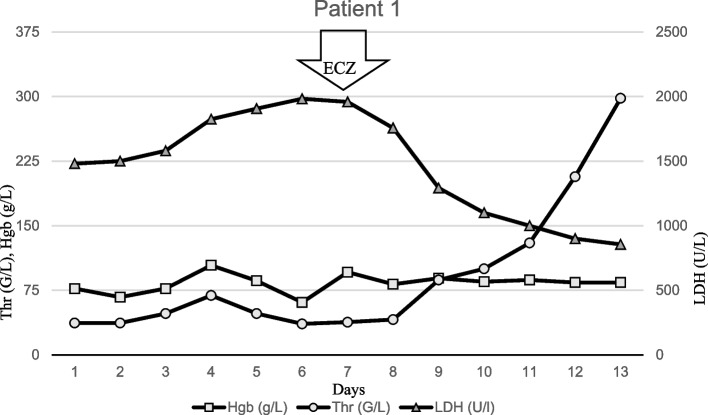
Timeline of patient 1 treated with ECZ. The first dose of ECZ was given on Day 7. 3 days after the first administration of ECZ, the platelet count increased (from 38 G/L to 100 G/L without transfusion), the LDH activity significantly decreased (from 1959 U/L to 1100 U/L), diuresis increased, and dialysis was unnecessary. Abbreviations: Hgb, hemoglobin; Thr, thrombocyte; LDH, lactate dehydrogenase; ECZ, Eculizumab

A 2.5 year old female child, who was well previously, was admitted to our pediatric intensive care unit (PICU) with loss of appetite for 5 days, vomiting, loose stools (with no blood or mucus), abdominal pain, petechiae, orbital and limb oedema, without fever. Laboratory tests on admission were typical for TMA (Table 1). Direct Coombs test and virulence marker used to confirm EHEC from a stool sample were negative. Despite the adequate supportive therapy, significant progression was seen as the patient became oliguric over the next 4 days, developed general oedema, her kidney function deteriorated with worsening TMA related hemolysis. Based on the negative stool E.coli test (verotoxin negative) and the suspicion of aHUS due to an uncertain infectious and unknown family history, ECZ was administered on the 7th day. Consequently, within 3 days, dramatic improvement was seen in TMA related hematological activity, diuresis improved and dialysis became unnecessary (Fig. 1). Given the favorable response to ECZ, it was repeated once 7 days later. In the following 2 weeks, her condition improved dramatically. Eventually, repeated stool bacteriological tests confirmed EHEC. Immunoserological tests (C3, C4, CH50) did not indicate dysregulation of the alternative pathway of the complement system, while the level of the activation complex of the terminal pathway increased markedly, indicating ongoing complement activation (Fig. 1). Subsequent genetic tests confirmed MCPggaac risk haplotype in homozygous form. The patient was discharged with an eGFR value of 63 ml/min/1,73m^2^ and physiological blood count parameters (Table 2).

**Table 1:** Initial presentation, laboratory values and complement diagnostic on admission ​​of patients with infection-associated HUS treated with ECZ

**Patient**	Patient 1	Patient 2	Patient 3
**Initial presentation**			
Age (months)	31	28	125
Gender	F	F	M
Weight (kg)	15	11.6	29
Height (cm)	103	90	136
Presenting complaints	vomiting, oedema	pneumonia, DIC	fever, watery diarrhea, abdominal pain
Neurological symptoms	No	No	Encephalopathy, seizure (tonic), hemiparesis
Onset of neurological symptoms from illness	No	No	Day 4
Haemoglobin (g/L)	87	62	98
Thrombocytes (G/L)	37	6	60
Creatinine (μmol/L)	211	152	730
Urea (mmol/L)	27.4	27.3	53.7
LDH (< 500 U/L)	1481	6938	3517
Fragmentocytes	Yes	Yes	Yes
C-reactive protein (< 2.2 mg/L)	5.5	201	23.6
PCT (< 0.5 ug/L)	N.A	108.19	70.63
Prothrombin time (9.3 s)	8.1	8.6	9.5
International normalized ratio	0.93	0.88	1.02
Activated partial thromboplastin time (28.6 s)	34.2	85.6	32.5
Thrombin time 16.3 s)	20.3	12.9	18.5
Direct Coombs test	Negative	Positive	Negative
Type of HUS	STEC-HUS	SP-HUS	STEC-HUS
Stool culture	E.coli stx1/2	Negative	E.coli stx1/2
Blood culture	Negative	Pneumococcus	Negative
**Complement diagnostic at admission**			
ADAMTS13 metalloproteinase activity (67–151%)	90	11	27
CH50 (48–103 CH50/ml)	59	0	47
APH50 (70–125%)	113	48	82
C3 (0.9–1.8 g/L)	1.13	0.53	0.9
C4 (0.15–0.55 g/L)	0.17	0.1	0.11
CFH (250–880 mg/L)	366	175	246
CFI (70–130%)	102	38	90
CFB (70–130%)	97	72	10
C1q (60–180 mg/L)	38	88	113
sC5b-9 (110–252 ng/mL)	258	2459	845
Haptoglobin (0.3–2 g/L)	0.02	0.2	0.15

*Abbreviations*: *F* Female, *M* Male, *LDH* Lactate dehydrogenase, *PCT* Procalcitonin, *HUS* Hemolytic uremic syndrome, *SP-HUS* Streptococcus pneumoniae-associated HUS, *STEC-HUS* Shiga toxin-producing Escherichia coli-HUS, *AP* Complement activation, *APH50* Alternative pathway total complement activity, *CFB* Complement factor B, *CFH* Complement factor H, *CFI* Complement factor I, *CH50* Total complement activity, *SC5b-9* terminal Complement complex

**Table 2:** Clinical parameters and outcome data of patients with infection-associated HUS treated with ECZ

**Patient**	Patient 1	Patient 2	Patient 3
**Clinical parameters**			
Time from HUS Dg to dialysis (days)	N.A	1	1
Type of dialysis	N.A	CVVHDF	CVVHDF
Duration of dialysis (days)	0	10	13
Time to PLT normalization from administration of ECZ (days)	3	1	1
Time to LDH normalization from administration of ECZ (days)	7	7	5
Time from HUS Dg to ECZ treatment (days)	7	6	9
ECZ doses	2	2	2
Proven bacterial infection within 6 weeks of ECZ administration	No	No	No
Proven viral infection within 6 weeks of ECZ administration	No	Yes	Yes
Plasma exchange	No	No	4
Need for transfusion	Yes	Yes	Yes
No. of RBC transfusion (unit)	3	7	9
No. of PLT transfusion (unit)	4	20	16
Ventilation support	No	HFNC	MV
Catecholamines use	No	No	Yes
Antibiotic use	No	Yes	Yes
Pleural effusion	No	Yes	Yes
Pleural effusion drainage	No	Yes	Yes
Throrascopy	No	Yes	No
Neurologic symptoms	No	No	Yes
Gastrointenstinal complications	No	No	Yes
**Outcome**			
Death	No	No	No
Length of ICU stay (days)	7	17	14
Length of stay total in hospital (days)	14	24	25
Renal function recovery (days)	3	10	13
eGFR at exmission (ml/min/1.73 m2)	63	67	81
Hypertension	No	No	Yes
Proteinuria	Yes	Yes	Yes
Neurologic symptoms at exmission	No	No	Yes
**Follow-up examination (3 months)**			
eGFR (ml/min/1.73 m2)	120	130	120
Hypertension	No	No	Yes
Proteinuria	No	No	Yes
Neurologic symptoms	No	No	No

*Abbreviations*: *HUS* Hemolytic uremic syndrome, *CVVHDF* Continuous venovenous hemo-diafiltration, *PLT* Platelet, *LDH* Lactate dehydrogenase, *RBC* Red blood cell, *HFNC* High flow nasal cannule, *MV* Mechanical ventilation, *ICU* Intensive care unit

### Patient 2

**Fig. 2:**
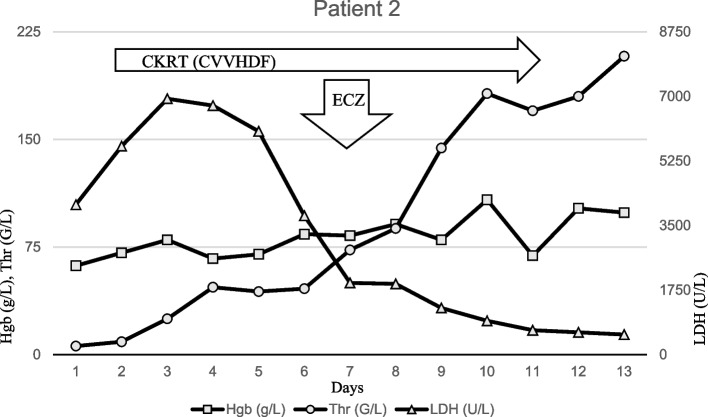
Timeline of patient 2 treated with ECZ. The first dose of ECZ was given on Day 7. 3 days after the first administration of ECZ, the platelet count increased (from 73 G/L to 182 G/L without transfusion), LDH activity significantly decreased (from 1946 U/L to 918 U/L), diuresis increased, dialysis was stopped after a total of 10 days of treatment. Abbreviations: Hgb, hemoglobin; Thr, thrombocyte; LDH, lactate dehydrogenase; CKRT, continuous kidney replacement therapy; CVVHDF, continuous veno-venous hemodiafiltration; ECZ, Eculizumab

A 28 month old female child presented at our institute with a 4-day history of pneumonia-associated fever, vomiting, shortness of breath and oliguria in association with gross hematuria. Her previous history was uneventful, she received the mandatory vaccinations, including pneumococcal polysaccharide vaccine containing 13 serotypes. The admission lab tests were characteristic of TMA with elevated inflammatory markers and evidence of disseminated intravascular coagulation (DIC). Direct Coombs test was positive (Table 1). The radiological examinations confirmed right-sided pneumonia with pleural effusion. Overall, the results of the clinical, laboratory and radiological examinations corresponded with the diagnosis of invasive pneumococcus infection and an associated SP-HUS which was supported by hemoculture positivity for Streptococcus pneumoniae. Her management included combined antibiotic treament and supportive therapy (transfusion, intravenous immunoglobulin, respiratory support), which was supplemented from day 2 with continuous kidney replacement therapy (CKRT) due to oliguric AKI, significant fluid overload and metabolic acidosis. Due to worsening respiratory distress with increasing fibrinopurulent chest fluid, critical thrombocytopenia, and coagulopathy, primary video-assisted thoracic surgery (VATS) and chest drainage were performed. Due to ongoing significant hematological activity, uncontrolled complement activation (reduced C3, C4 and CH50 levels and alternative pathway activity, extremely elevated terminal pathway activation markers) (Table 1), administration of ECZ was initiated on Day 7 with excellent clinical response (Fig. 2). ECZ was discontinued after two doses of ECZ as kidney function improved, the child recovered without residual symptoms. No genetic abnormalities were detected. She was discharged with an eGFR value of 67 ml/min/1.73m^2^ and physiological blood count parameters (Table 2).

### Patient 3

**Fig. 3:**
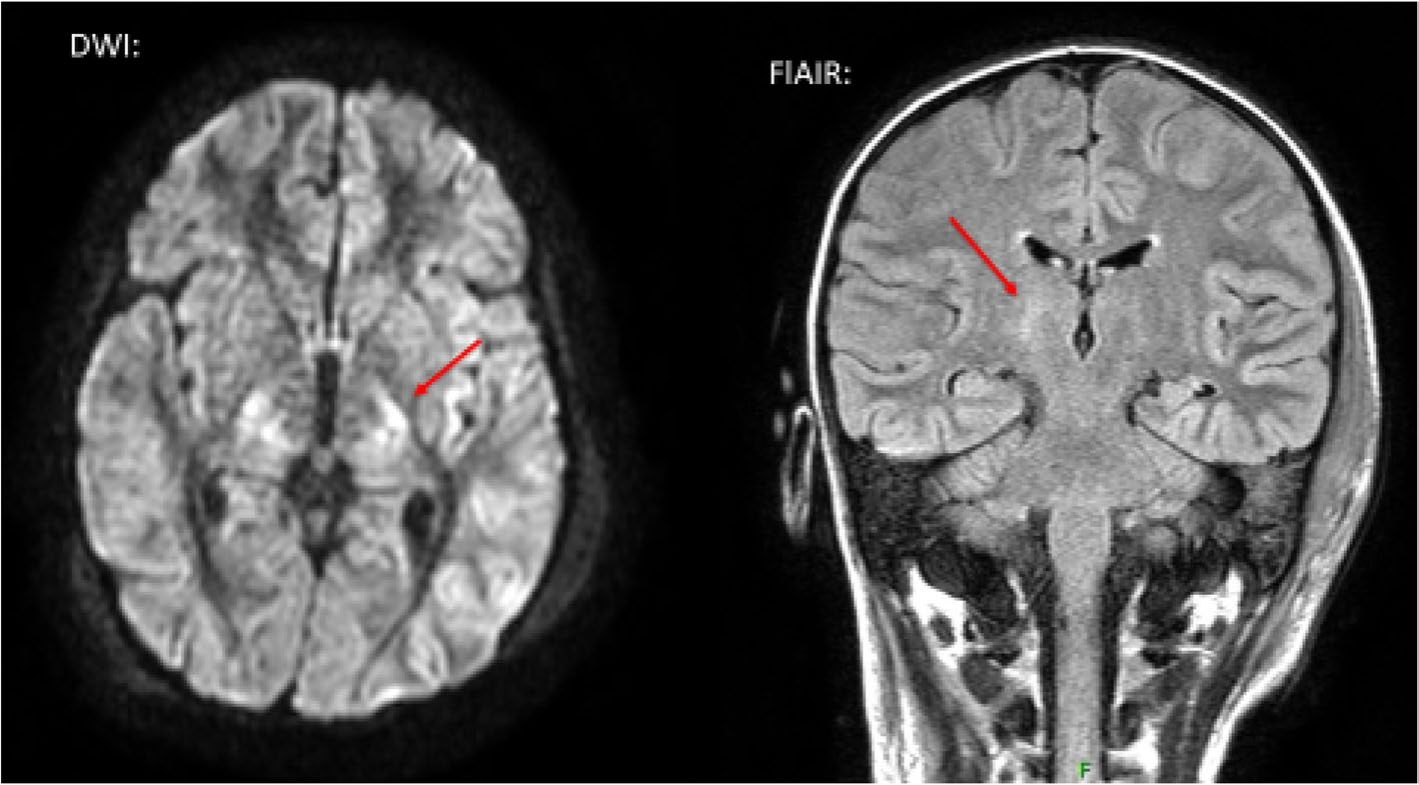
MRI of patient 3. On the MRI (FLAIR and DWI) images, a symmetric, hyperintense, banded abnormality can be seen in the area of ​​both thalamus, on the basis of which the abnormality may correspond to Percheron arteriopathy (arrow). The Percheron artery is a rare anatomical variant, in which both lateral vessels originate from the same main trunk, and its injury (vasculitis, thromboembolism) causes a symmetrical deviation. The symptoms, such as fluctuating cognitive impairment, aphasia, memory impairment, and right-sided motor symptoms were all detectable in our 3rd patient and corresponded to the abnormality seen on the MR image

**Fig. 4:**
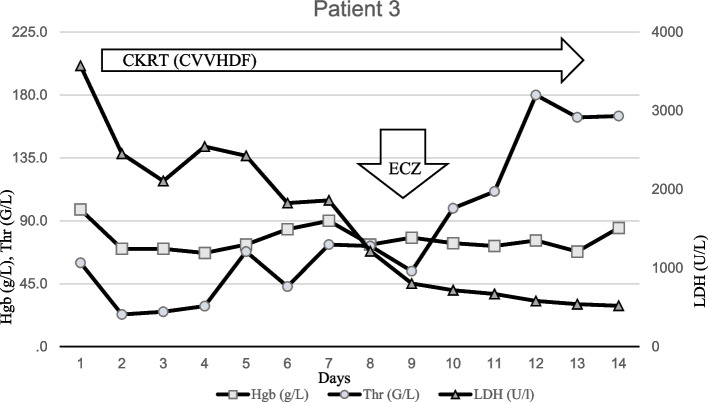
Timeline of patient 3 treated with ECZ. The first dose of ECZ was given on Day 9, after which sudden and dramatic improvement was seen in hematological activity resulting in no need for further transfusions and quick normalization of hematological parameters. 3 days after the first administration of ECZ, the platelet increased (from 54 G/L to 180 G/L without transfusion) and LDH activity decreased (from 803 U/L to 581 U/L). Dialysis was stopped after a total of 13 days of treatment. Abbreviations: Hgb, haemoglobin; Thr, thrombocyte; LDH, lactate dehydrogenase; CKRT, continuous kidney replacement therapy; CVVHDF, continuous veno-venous hemodiafiltration; ECZ, Eculizumab

The previously healthy 10-year-old boy was admitted with complaints of watery, non-bloody diarrhea, abdominal pain, and fever lasting for 4 days. The laboratory tests performed at the time of admission confirmed TMA with leukocytosis, markedly elevated inflammatory values (Table 1). In addition to the clinical picture, the EHEC positivity confirmed by the PCR test from stool sample suggested the diagnosis of STEC-HUS. The critically ill patient required antibiotic treatment (meropenem) for severe abdominal symptoms, presumed sepsis caused by bacterial enterocolitis. Despite the administration of intravenous fluids and diuretics, on the 2nd day after admission CKRT was started due to prolonged anuria, volume overload and polyserositis (mainly pleural effusions). Despite CKRT, progressive bilateral pleural effusion developed requiring bilateral pleural drainage, and non-invasive ventilation therapy. Due to severe hematological activity, regular transfusions were given. On the 4th day of treatment, fluctuating and later worsening consciousness was noticed. Despite plasma exchange (PLEX) treatment severe extrarenal symptoms (polyserositis, CNS involvement) including neurological symptoms progressed (tonic–clonic convulsion, aphasia, right-sided hemiparesis). Cranial MRI revealed a symmetric, hyperintense, banded abnormality on FLAIR and DWI images in the area of ​​the thalamus on both sides, which corresponded to Percheron arteriopathy (Fig. 3). Serological studies obtained earlier confirmed the global abnormal activation of the complement system with low level of complement factors (C3, C4, CH50) and a markedly elevated terminal pathway activation (sC5b-9) (Table 1). As a consequence of an unsatisfactory clinical response to supportive therapy and plasmapheresis the use of ECZ was indicated. ECZ was administered on the 9th day, after which sudden and dramatic improvement was seen in TMA related hematological activity with no further need for transfusions (Table 1). Dialysis was stopped after a total of 13 days of treatment (Fig. 4). A gradual improvement in neurological symptoms was observed. The patient was discharged with an eGFR value of 81 ml/min/1.73m^2^ and physiological hematological parameters. During complex rehabilitation, his neurological symptoms fully regressed (Table 2). Subsequent genetic tests confirmed that the patient carries the MCPggaac risk haplotype in homozygous form.

## Discussion and conclusions

In the presented case series we report about the clinical course and outcome of three children with severe HUS. In all three cases the typical triad of TMA related hemolytic anemia, thrombocytopenia and AKI were observed with the activation of the complement system. The complement system can be initiated depending on the context by three distinct pathways – classical, lectin, and alternative (AP), each leading to a common terminal pathway activation. During severe infection classical and lectin have a critical role in pathogen recognition and initiation of complement cascade. However, the AP assures more than 80% of the terminal complement activity during the pathogen recognition process. The clinical presentation showed substantial differencies and variations among the cases (2 STEC-HUS and 1 SP-HUS) highlighting the difficulties of diagnosing and managing pediatric TMA cases. Initial management was provided on the basis of best supportive care in each case [20, 22] (KDIGO). Eventually, all three patients received C5-complement inhibitor (ECZ), either due to the suspicion of aHUS case or due to the progressive course of the disease with prolonged CKRT requirement, persistent hematological activity, central nervous system (CNS)/multiorgan involvement and evidence of complement activation. In the first patient (Patient 1), in addition to the negativity of the first stool for verotoxin, based on the progression observed in the clinic (worsening kidney function, prolonged anuria and severe hematological activity), ECZ therapy was started with the suspicion of aHUS. Rapid improvement was observed within 72 h including hematological parameters and diuresis returning to normal. Interestingly, repeated test (verotoxin PCR from stool) in a reference laboratory finally revealed STEC-HUS. The serum complement parameters showed only borderline activation with normal C3, C4, CH50 levels and mild elevation of sC5b-9. Atypical HUS requires an immediate intervention and decision to start ECZ is recommended at an early stage [23, 24]. The other patient with STEC-HUS (Patient 3) had severe extrarenal manifestations including CNS involvement and polyserositis. The Percheron artery is a rare anatomical variant, in which both side vessels originate from the same main trunk, and its injury (vasculitis, thromboembolism) causes a symmetrical deviation. Symptoms such as fluctuating cognitive impairment, aphasia, and memory impairment were detectable in our patient with hemiparetic symptoms on the right side corresponding to the deviation seen in the MR image [25, 26] (Fig. 4). In agreement with our current institutional protocol we performed a series of plasma exchange (PLEX) with fresh frozen plasma (FFP) (4 sessions) without detectable clinical improvement. The complement profile before the administration blood products and plasmaferesis showed global activation of the complement system with markedly decreased C3, C4, CH50 levels and highly elevated sC5b-9 value. Worsening CNS symptoms triggered the use of ECZ treatment. ECZ treatment resulted in remarkable improvement in the kidney function parameters (Fig. 3) as well as in CNS complications and hematological activity (Table 2). Cognitive dysfunctions, hemiparetic symptoms and aphasia were quickly resolved. A rapid improvement in both clinical and laboratory parameters correlate well with outcome of similar cases reported by others with ECZ treatment of STEC-HUS associated with severe CNS complications [12, 16, 27–29].

Our SP-HUS patient’s (Patient 2) clinical and laboratory findings corresponded with sepsis, bilateral pleuropneumonia, MOF, DIC and parallel TMA with unusually high level of LDH, deep thrombocytopenia (PLT: 7 G/L) and severe AKI. Severe global activation of the complement system and an extended endothelial damage was detected with characteristic alterations in TMA related values (ADAMST13, C3, C4, CH50, sC5b-9) (Table 1). We think that a „biphasic” change took place in biochemical and hematological parameters as bloodstream infection-pneumonia-sepsis and complement-associated TMA occured. Treatment of sepsis/DIC and pleural effusion (antibiotic treatment, IVIG, FFP and VATS) may have resulted in significant improvement in corresponding parameters (sepsis-induced TMA part improved: early LDH decrease, modest elevation of PLT count (from 7 G/L to about 30 G/L (Fig. 2)), however complement-mediated TMA related to extensive endothelial damage (neuraminidase related component) was still active. Global and extreme activation of the complement system (sC5b-9: 2759 ng/ml) indicated ECZ treatment in parallel with dialysis-dependent state, persisting severe AKI, prolonged ventilatory support, and severe hematological activity. Dialysis dependence, polyserositis, hemosupportation and prolonged anuric state may also be factors in the reduced survival rate, as the estimated mortality of SP-HUS may reach 10%. Literature data about the use of C5-inhibitor in pediatric SP-HUS patients ECZ is scarce. Only few case reports and case series reports recount successful use of ECZ particularly in desperate clinical situations (severe CNS involvement or progressive and therapeutic resistant cases) where dysregulation of the complement pathway was presumed to play a central role in the disease pathophysiology [19, 21, 30, 31]. A Czech study published in 2023 reported the use of ECZ in 4 cases out of 7 SP-HUS patients, with no evident advantage of ECZ in their cohort [31].

Determination of the complement profile before the administration blood products or plasmaferesis (3rd case) has two implications for these cases: first, decreased classical pathway activity and components indicate ongoing inflammation and infection, whereas second, decrease of C3, CFB and CFH indicate the extent of alternative pathway amplification and consumption. If both are parallel present, one may conclude (just like for cases 2, 3) that infection triggered classical pathway activation led to alternative pathway overactivation, and complement may be associated with the pathogenesis. Beyond TMA related microangiopathic cell damage, in all three cases the core element of the pathomechanism was the predominant activation of the complement alternative pathway with consumptive decrease in complement factor C3 and C4 (Patient 2 and 3) and increased sC5b-9 levels (Patient 1, 2, 3) (Table 1). Both detailed complement activation profile and genetical analysis were obtained in all three cases. Previous experimental and clinical observations proved that dysregulation of the complement alternative pathway could be a major pathogenetic event in both STEC-HUS and SP-HUS [18, 19, 32]. Even though ECZ received both FDA and EMA approval for the treatment of aHUS [33, 34], its use in STEC-HUS or SP-HUS remained elusive [17, 27, 35]. A recently published randomized controlled trial (RCT) about C5 inhibitor (ECZ) treatment in STEC-HUS found no convincing evidence in the short-term outcome as measured in time of CKRT need and eGFR in the acute phase, however convincing evidence was shown for the long-term benefit (significantly better eGFR in the treated group) of ECZ treatment in STEC-HUS [17]. Surprisingly good outcome was reported in case series of STEC-HUS associated with CNS complications, where ECZ indication was solely based on the clinical situation (TMA activity and severity of CNS involvement) [12, 27, 29]. On the basis of previous experience with aHUS, early administration of ECZ has been associated with better long-term kidney outcome [11, 23, 24]. In our cases evaluation of complement activation markers was helpful, even though the disease course and clinical presentation were the major determinants in decision making. Both parental consent and the Hungarian National Drug Administration license was obtained before the administration of ECZ. In agreement with available guidelines patients received antibiotic treatment or prophylaxis for the safe administration of ECZ and vaccination against all Neisseria strains in the earliest possible time [36, 37].

We believe that all three patients responded impressively well to ECZ. Global activation of the complement system (Patient 2 and 3) may correspond with more extensive activation process that is not limited to the alternative pathway. In case 2 with SP-HUS, sepsis, DIC and neuraminidase-induced extensive endothelial damage while in case 3 STEC-related cell destruction augmented by inevitable antibiotic treatment may explain enhanced and global activation of the complement system. Consequently, inhibition of C5 may have been a valid strategy to blunt ongoing complement-mediated pathological events [12, 32, 38].

Previous publications did not present convincing data about the advantage of ECZ use in STEC-HUS, however some benefits of long-term outcome were reported [17, 27, 35]. In our cases both quick recovery and excellent outcome after ECZ treatment convinced us that in similar clinical situtations (CNS involvement and/or severe multiorgan dysfunction) we would consider using ECZ again. We emphasize that complement activation data in hand may help clinical decision.

Limitation of our study: Due to the lack of control group our case series could not provide convincing evidence for the use of ECZ in infection-related HUS patients. Randomized controlled clinical trials and/or a well-designed registries with comparable cases of infection-associated HUS would help to evaluate the real short- and long-term benefits or risks of ECZ use in similar cases.

### Adverse events with the administration of ECZ

Administration of ECZ is known to increase the risk of infections [37]. Therefore, antibiotic prophylaxis and vaccination against Neisseria species are recommended before the administration of the drug (see Soliris (eculizumab) package insert). Less is known about the incidence of other potential infective agents [39]. In two patients symptomatic cytomegalovirus (CMV) infection was detected with relatively higher copy number (Patient 2 and 3) after the 2nd dose of ECZ. In both cases specific antiviral therapy was indicated. The incidence of CMV infection is unknown in ECZ treated pediatric patients. Our cases highlight the importance of checking on CMV status upon ECZ treatment since C5-inhibiton in a sense is an immunocompromised state and an ongoing CMV-disease may compromise clinical improvement [40, 41].

To our best knowledge, genetical analysis is not part of the routine diagnostics in infective HUS [36]. Even though infection is the major trigger of disease manifestations, current literature data suggest that risk haplotypes of complement regulatory genes are occasionally detected in cases of HUS. Indeed, in two patients (Patient 1 and 3) we detected MCPggaac risk haplotype in homozygous form, which may increase the chance of a more severe HUS/TMA manifestation [42]. We did not consider long-term ECZ treatment in these cases, and we do not expect and have not experienced recurrence as yet.

We believe that detailed complement activation data, when it is available in a short turnaround time, is helpful in deciding about the use of ECZ and may in the future be an important part of the diagnostic panel. Timing of ECZ administration depends on many variable factors including parental consent or institutional and other official approvals. We report excellent outcome of our patients suggesting that the time-window for ECZ treatment is not yet defined [40, 43].

### Summary

The use of complement C5 inhibition is still controversal in the treatment of infection-associated HUS types. Currently, the standard of care in infection-associated HUS types is mainly supportive. In selected HUS cases with CNS involvement and other severe extrarenal manifestations C5-inhibitor treatment (ECZ) may be used. Parental consent and/or institutional and other official approvals are all necessary for off-label use of ECZ. Early use of ECZ may improve both short-term and definitely long-term outcome especially in cases where complement system (AP) is overactivated. Detailed complement activation profile, particularly sC5b-9 is helpful to indicate ECZ administration. Proper antibiotic prophylaxis/treament and vaccination against Neisseria species are recommended for safer use of ECZ. Monitoring of bacterial, viral infection including CMV is necessary for patient’s assessment. Well-designed multicenter RCTs would help gain further evidence to support the benefit of complement inhibition in the treatment of severe pediatric HUS with multiorgan involvement.

## Data Availability

Dataset analyzed during the study represent patient’s data available in their medical documentation and the electronic patients’ database (MedSolution, UDMed) tracking system of the University of Debrecen for authorized personnel. Petra Varga, the first author can be contacted for additional data request (varga.petra@med.unideb.hu).

## Abbreviations

aHUS: Atypical hemolytic uremic syndrome
AKI: Acute kidney injury
AP: Alternative complement pathway
APH50: Alternative pathway total complement activity
BMI: Body mass index
BW: Body weight
CFB: Complement factor B
CFH: Complement factor H
CFHR5: Complement factor H-related protein-5
CFI: Complement factor I
CH50: Total complement activity
CKRT: Continuous kidney replacement therapy
CMV: Cytomegalovirus
CNS: Central nervous system
DGKE: Diacylglycerol kinase epsilon
DIC: Disseminated intravascular coagulation
ECZ: Eculizumab
EMA: European medicines agency
FDA: US Food and drug administration
FFP: Fresh frozen plasma
HUS: Hemolytic uremic syndrome
PICU: Pediatric intensive care unit
PLEX: Plasma exchange therapy
RCT: Randomized controlled trial
sC5b-9: Solubilis terminal complement complex
SP-HUS: Streptococcus pneumoniae-associated HUS
STEC-HUS: Shiga toxin-producing Escherichia coli-HUS
THBD: Thrombomodulin
TMA: Thrombotic microangopathy
VATS: Video-assisted thoracic surgery
